# Exo-metabolome profiling of soybean endophytes: a road map of antagonism against *Fusarium oxysporum*

**DOI:** 10.1128/msystems.00927-25

**Published:** 2025-10-01

**Authors:** H. Tariq, C. Viau, S. Subramanian, A. Geitmann, D. L. Smith

**Affiliations:** 1 Department of Plant Science, Macdonald Campus, McGill University 5620, Sainte Anne de Bellevue, Québec, Canada; 2 Institute of Parasitology, Macdonald Campus, McGill University 5620, Sainte Anne de Bellevue, Québec, Canada; Pacific Northwest National Laboratory, Richland, Washington, USA

**Keywords:** antifungal metabolites, *Bacillus*, *Fusarium oxysporum*, LC-MS/MS, soybean, untargeted metabolomics

## Abstract

Soybean is one of the world’s most valuable agricultural crops. The phytopathogen *Fusarium oxysporum* compromises the growth of soybean and decreases its yield. Controlling this pathogen with fungicides is environmentally harmful. As with other species, soybean plants are associated with beneficial bacterial communities that contribute to plant health. Based on 16S rRNA sequencing, the present study identifies three important bacterial strains from this community: *Bacillus*-HT1, *Bacillus*-HT2, and *Bacillus*-HT3. Screening of these bacteria against *F. oxysporum* of soybean showed that *Bacillus-*HT1 and *Bacillus-*HT2 are antagonistic against this pathogen. The overall changes in fungal structure caused by biocontrol bacteria were visualized using scanning electron microscopy. An untargeted metabolomics approach based on liquid chromatography–tandem mass spectrometry was employed to uncover the bacterial secondary metabolites underlying this antagonistic activity. Based on MS2 fragmentation analysis and annotation, *Bacillus-*HT1 and *Bacillus*-HT2 potentially produce significant amounts of putative antifungal compounds, whereas *Bacillus*-HT3 lacks these. This absence of relevant metabolites correlates with this strain’s inability to inhibit *F. oxysporum* fungal growth. Among the putative antifungal metabolites produced by *Bacillus-*HT1 and *Bacillus*-HT2 are bacillibactin and surfactin C. *Bacillus*-HT1 also produces significant amounts of fusidic acid and kynurenine, while *Bacillus-*HT2 produces significant amounts of putative antifungal fatty acids. These findings suggest that *Bacillus*-HT1 and *Bacillus-*HT2 have the potential to be developed into effective biocontrol agents, reducing chemical fungicides in crop cultivation, improving plant health and yield, thus contributing to the protection of biodiversity and soil health in the long term.

**IMPORTANCE:**

Modern agricultural practices depend heavily on synthetic fertilizers and pesticides, which are major contributors to greenhouse gas emissions, groundwater pollution, and disruptions in agroecosystem dynamics. These challenges underscore the pressing need for sustainable alternatives that maintain crop productivity while minimizing environmental impact. Here, we investigate the use of antifungal-producing biocontrol agents as a microbial-based strategy to suppress pathogenic fungi in soybean cultivation. By harnessing the metabolic capabilities of beneficial microbes, this approach offers a promising path toward environmentally responsible crop protection, with implications for future food security and sustainable agricultural systems.

## INTRODUCTION

Soybean (*Glycine max* [L.] Merr.) is a leguminous crop plant in the family Fabaceae that has high nutritional value and contains vitamins, an edible oil concentration of about 20%, and a protein content of 20–25% (1). One of the primary pests affecting soybean yield is *Fusarium oxysporum*, a globally distributed soil-borne fungal pathogen that exhibits facultative parasitic behavior and causes soybean root rot. *F. oxysporum* colonizes the vascular system of plant roots, produces a variety of virulence factors, and causes plant cell death. *Fusarium* is one of many biotic stresses that have a significant impact on agricultural productivity. Food production has become reliant on fertilizers and pesticides that contribute to global warming. As a result, the emission of greenhouse gases and groundwater contamination leads to disturbance in agroecosystems (2, 3). Alternative solutions to promote sustainable crop production and future food security are therefore important, and here we explore the possibility of developing biocontrol methods to reduce the effects of a detrimental fungus in the context of soybean cultivation.

The roots of soybean secrete metabolites that act as signaling compounds, allowing them to interact with their phytomicrobiome. These metabolites include signaling molecules such as isoflavones and strigolactones. The rhizosphere includes nitrogen-fixing bacteria such as *rhizobia*, other plant growth-promoting bacteria, and arbuscular mycorrhizal fungi (4). Plant growth-promoting bacteria colonize the soybean root, reside inside tissues, and aid plant growth through either or both distinct mechanisms: (i) direct promotion of plant growth and enhancement of resilience to stress and (ii) antagonistic action against plant pathogens such as *F. oxysporum*. Several root-secreted metabolites of soybean attract beneficial bacteria that have the potential to interact with the host plant to increase its yield and improve plant resistance to biotic and abiotic stresses (5, 6). Studying the microbiota of soybean is particularly interesting since it is a model plant that attracts beneficial members of the microbiome using its root exudates. The microbiome associated with plants is categorized as plant growth-promoting rhizobacteria that reside in the soil and are directly in contact with roots and can promote plant growth (7). Some rhizosphere bacteria can penetrate the root surface and colonize internal plant tissues; they are termed endophytes. Microbes can benefit plant growth by fixing nitrogen, solubilizing inorganic soil phosphorus, increasing nutrient availability, sequestering iron by producing siderophores, and chelating the iron to make it available to plants (8, 9).

Omics technologies, in combination with bioinformatics, help uncover the mechanisms of action and interactions between endophytes and plants for improved plant growth and control of the phytopathogens (10, 11). Secondary metabolites are organic compounds with complex structures that, among other things, mediate intercellular communication, enabling bacteria to survive in adverse conditions and have a wide variety of biological properties (antibacterial, antifungal, antiviral, phytohormone, and signaling compounds) (12–14). Microbial compounds can have profound effects on plant growth promotion, disease resistance, and pathogen growth inhibition, and thus mediate bacterial effects on high-yield crop production (15, 16). To determine the mechanism of plant growth promotion properties of beneficial bacteria, it is necessary to explore bacterial exo-metabolic profiles. In the present study, tandem mass spectrometry (MS/MS) is used for high-throughput screening due to its increased sensitivity in metabolomics. The use of mass spectrometry can reveal elemental compositions, mass-to-charge ratios (*m*/*z*), isotopic patterns, abundances, and fragmentation patterns of molecules (17, 18). MS/MS spectra can be analyzed without any prior knowledge of the chemical composition by examining thousands of compounds (19, 20). Putative metabolite identification is based on the comparison of one or more molecular properties—typically accurate mass, MS/MS fragmentation patterns, or retention time—rather than direct confirmation using an authentic chemical standard analyzed under identical experimental conditions. The putative metabolite identification approach relies on matching experimental data to entries in reference databases that contain chemical structures or spectral information. Because the data used for comparison are often generated across different laboratories and obtained using varying analytical platforms and methods, the identification remains tentative (21–25). Somperformedf the common databases used for mass spectrometry are NIST, MassBank, the Golm Metabolite Database, METLIN, MoNA, MS-DIAL, and HMDB (26–31).

The primary aim of this study is to enhance our understanding of the bacterial endophytic diversity present in soybean plant roots. Furthermore, we seek to isolate and characterize novel endophytic bacterial strains that exhibit plant-beneficial properties by inhibiting the growth of fungal pathogens. We hypothesized that under optimal growth conditions, the endophytic bacterial strains associated with soybeans produce antifungal compounds in their exo-metabolomes that inhibit the growth of *F. oxysporum* in soybeans. By measuring the relative abundance of these putative bioactive compounds through untargeted metabolomics, we aim to identify the novel bacterial endophytes that play a crucial role in enhancing soybean resistance against fungal pathogens, thereby contributing to sustainable agriculture and improved crop health

## MATERIALS AND METHODS

### Sample collection

Soybean roots were collected from plants grown in the field in Emile A. Lods Agronomy Research Center, Macdonald Campus, McGill University, Sainte-Anne-de-Bellevue (Québec, Canada), a suburb on an island located at 45°24′22″ N, 73°56′44″ W and with an average elevation of 20.87 m above sea level in southwestern Québec. In the area, precipitation is distributed throughout the year, with 932 mm of rainfall per year. With an average annual temperature of 6.3°C, the climate is cold temperate. In July, average temperatures reach 20.7°C, while in January, they reach −9.7°C. The root systems of soybean were sampled on July 2023 by excavating the roots to a depth of 15–30 cm. Root samples collected were immediately wrapped in plastic bags, transported to the laboratory, stored at 4°C, and processed the following day.

### Isolation of endophytes

Surface sterilization of root samples was performed as described in Qin et al. (32) with some modifications. After brushing fresh roots, tap water was used to thoroughly rinse them to remove adhering soil and attached epiphytic bacteria. Any visibly damaged tissue was removed. After several rinses in ddH_2_O, until the water was clear, the roots were cleaned with Sparkleen 1 (Fisherbrand; sodium carbonate, 10%–25%; sodium dodecylbenzene sulfonate, 1%–10%; and non-ionic detergent 1%–10%) water solution (vol/vol), rinsed several times in ddH_2_O, and dried with sterile paper towels. Using sterile scissors, a pooled mixture of root samples was cut into 1- to 2 cm portions, transferred into separate sterile tubes, surface sterilized with 70% (vol/vol) ethanol for 10 min, rinsed three times with sterile ddH_2_O, and then shaken in commercial bleach (3% chlorine) for 5–10 min, followed by six changes in sterile ddH_2_O to remove the disinfectant. Two methods were used to confirm surface disinfection. An aliquot of ddH_2_O from the last wash was plated onto King’s B media (20 g proteose peptone no. 3, 10 mL glycerol, 1.0 g MgSO_4_, 1.5 g K_2_HPO_4_, pH 7.2), tryptic soy agar (casein peptone [pancreatic] 15 g, soy peptone [papainic] 5 g, sodium chloride 5 g, agar 15 g, final pH 7.3 ± 0.2 at 25°C), nutrient broth agar (8 g/L, Difco), and plates and examined for contaminant colonies after 3–7 days of incubation at 28°C. In the second step, surface-sterilized tissues were imprinted onto the agar, incubated at 28°C, and then assessed for microbial growth. Following sterilization, the samples were thoroughly dried under a laminar flow. Root fragments in 1 g samples were then macerated aseptically in phosphate-buffered saline (PBS) (pH 7.2) with a sterile mortar and pestle; the supernatants were diluted in 10-fold series in PBS to 10^−3^; and resulting aliquots of 100 µL of the first to third diluents were plated in triplicate onto agar solidified growth media to recover any bacterial endophytes present in the plant root tissue. The agar plates were incubated at 28°C for 3–5 days, after which colony-forming unit (CFU) per gram was determined. By single-colony streaking, morphologically different colonies were chosen and purified three times on the original medium, based on their rate of growth and their characteristics on the plates, such as size, shape, texture, and color. To maintain the colonies, isolates were sub-cultured and then frozen in the appropriate growth medium, containing 50% glycerol, at −80°C until use.

### Characterization of traits

Endophytic bacterial isolates were characterized based on colony morphology and molecular phylogeny. Three isolates (HT1, HT2, and HT3) were selected for further characterization.

### 16S rRNA gene amplification and sequencing

Standard polymerase chain reaction (PCR) was used to taxonomically identify, to the species level, the selected bacterial isolates from the primary screening assay. Sanger sequencing was carried out at Genome Québec Innovation Center to analyze the 16S rRNA region of the bacterial isolates. Target regions were amplified and sequenced using universal primers 27F (5′-AGRGTTYGATYMTGGCTCAG) and 1492R (5′-AYCTCACGRCACGAGCTGAC). The 16S rRNA gene encodes genetic information that can be used to identify and characterize bacterial strains. The samples were diluted in water at a ratio of 1:10 for analysis. PCR was performed using the Fast HotStart enzyme from Kapa Biosystems from diluted samples. Sanger sequencing using Applied Biosystems’ Big Dye Terminator v.3.1 was performed on the purified PCR products. A Sanger sequencing kit (Big Dye Terminator v.3.1) was used to analyze purified PCR samples (manufactured by Applied Biosystems). Following an initial denaturation at 96°C for 1 min, 25 cycles of denaturation at 96°C for 10 seconds, annealing at 50°C for 5 seconds, extension at 60°C for 4 min, and the final extension at 4°C were performed. The resulting sequencing reactions were analyzed using an Applied Biosystems 3730xl DNA Analyzer. National Center for Biotechnology Information Basic Local Alignment Search Tool (https://blast.ncbi.nlm.nih.gov/Blast.cgi) was used to compare the sequences obtained with previously published 16S rRNA sequences.

### Antifungal property

Each bacterial strain was confronted with fungi in dual-culture petri plates in an *in vitro* confrontational assay (33). The *Fusarium oxysporum* of soybean was provided by Agriculture and Agrifood Canada. Mycelium plugs (5 mm) containing *Fusarium oxysporum* were placed in the center of petri plates with 20 mL of fresh potato dextrose agar. Bacterial cultures in their logarithmic growth phase were diluted to a concentration of 10^8^ CFU/mL in tryptic soy broth using a spectrophotometer reading at 600 nm. Inoculation of the bacterial cultures was conducted by adding two drops (5 µL each) at equal distances (30 mm) from the mycelium plugs, while control petri plates were not treated. Petri plates were kept at 25°C in the dark until the mycelium reached the edges of the control plates (3 days). Each combination of bacteria and fungi was replicated five times in petri plates, which were stacked randomly. The experiment was replicated twice. Changes in the morphology of *F. oxysporum* terminal hyphae in response to antifungal metabolites exuded by bacteria were assessed using scanning electron microscopy (SEM). Disks of 5 mm in diameter were collected at the edges of the fungal colony on each plate of the antagonism bioassay. SEM analyzes were conducted following Bozzola and Russell’s (34) protocol. After collection, the disks were immediately fixed in modified Karnovsky solution (2.5% glutaraldehyde, 2.5% formaldehyde in PBS buffer 0.05 M, pH 7.2, and CaCl_2_ 1.0 mM) for a minimum of 24 h with constant shaking at room temperature. The fixed disks were washed with PBS buffer three times for 10 min followed by washing in distilled water three times and dehydration in ethanol solution with increasing concentrations (25%, 50%, 75%, 90%, and 100%) with the last step of 100% ethanol repeated three times. The disks were dried in a critical point dryer (Leica EM-CPD). The disks were then placed on aluminum supports (stubs) and coated with a 4 nm layer of gold and palladium in a Leica ACE-200 sputter coater for morphological observation in a scanning electron microscope (Hitchai TM-1000). Micrographs were acquired at 15 kV with a backscattered electron detector at a working distance of 7 mm at room temperature. Scanning electron microscopy was conducted at the Multi-scale Imaging Facility at Macdonald Campus, McGill University.

### Metabolomics study of the cell-free supernatant

Bacterial metabolites were studied by growing isolated bacteria in tryptic soy broth. After the late exponential growth phase (48 h of growth), cultures were centrifuged at 4°C at 10,000 rpm (15,180 × *g*, SLA-1500) on a Sorvall Biofuge Pico (Mandel Scientific, Guelph, ON, Canada) for 10 min to separate bacterial cells from the supernatant. Further purification was conducted through vacuum filtration using a 0.22 µm filter to ensure that no cells were present in the cell-free supernatant (CFS). Twenty milliliters of CFS was kept in 50 mL falcon tubes at −80°C for 2 days followed by lyophilization (SNL216V freezing-dryer; Thermo Savant Co. Ltd., USA) for 3–5 days. The dried material was dissolved in 2 mL of 50% methanol in PBS buffer (pH 7.2) and kept overnight at −20°C. After 24 h, the solution was centrifuged at 80,000 rpm for 5 min. The supernatant was filtered using a 0.22 μm filter in a glass vial. One hundred microliter aliquots were submitted to the Institut de recherches cliniques de Montréal for analysis.

The liquid chromatography–mass spectrometry (LC-MS) analyses of the samples were done using Q-Exactive (Thermo Fisher Scientific, USA). Metabolite separation was performed on a Luna Phenomenex RP (15 × 2 mm, 3 µm particles) with security guard C18 cartridge (4 × 2.0 mm ID) installed in a Transcend II Ultimate 3000 UPLC system (Thermo Fisher Scientific). The mobile phase A was 100% H_2_O + 0.2% formic acid (FA) for positive mode and 100% H_2_O + 10 mM ammonium acetate for negative mode. Mobile phase B was 100% acetonitrile (ACN) + 0.2% FA for positive mode and 100% methanol for negative mode, and autosampler wash solution ACN/H_2_O (1/1, vol/vol) + 0.2% FA. The starting point of the gradient solution was 1% mobile phase B and increased up to 90% over 20 min. The flow rate was 300 µL/min, and the samples were thermostatic at 7℃ in the autosampler.

The Q Exactive mass spectrometer was operated in data-dependent acquisition mode using a HESI ion source. Full MS scans were acquired over an *m*/*z* range of 80–1,200 at a resolution of 70,000 (at *m*/*z* 200) in both positive and negative ionization modes. The 10 most abundant precursor ions were selected for fragmentation using higher-energy collisional dissociation (HCD) with a normalized collision energy of 35%, and MS/MS spectra were acquired in the Orbitrap. The isolation window was set to 2.0 *m*/*z*. The AGC target was set to 3e6 for MS scans and 1e5 for MS/MS scans, with maximum injection times of 100 and 50 ms, respectively. Product ion spectra were collected in profile mode at a normal scan rate. Dynamic exclusion was set to 4 seconds. A singly charged ion at *m*/*z* 255.23295 (negative mode) and *m*/*z* 371.10123 (positive mode) was used as a lock mass for internal calibration.

The source parameters were as follows: source voltages 3.6 kV (positive mode) and −3.0 kV (negative mode); sheath gas, 42 arbitrary units; auxiliary gas, 12 arbitrary units; sweep gas, 0 arbitrary units; capillary temperature, 320°C.

### Data analysis

Raw mass spectrometry data were converted and centroided with MSConvert (ProteoWizard v.3.0.24310) into mzML format for data pre-processing (35). The original data obtained from mass spectrometry were imported into MetaboAnalyst 6.0 software for spectral processing and database searching to qualitatively and quantitatively determine putative metabolites. Quality control was performed on the data to ensure the accuracy and reliability of the results. MetaboAnalyst 6.0 was used to optimize the parameters of the centWave algorithm (36, 37). CentWave was used to perform MS1 feature detection, and a peak table was generated. Both data polarities were treated separately. The multivariate analysis of PCA and partial least squares-discriminant analysis (PLS-DA) was performed to explore the total variation of the data and study the classification of the samples. The entire data sets were normalized with log transformation before analysis. MetaboAnalyst 6.0 was used for mummichog-based functional analysis, principal component *t*-test, and fold change analysis. Statistical significance was considered at *P* < 0.05, unless otherwise specified. The databases Kyoto Encyclopedia of Genes and Genomes (KEGG), HMDB, MoNA, MS-DIAL, GNPs, and MINE were used to annotate the identified MS1 peaks. The peaks that changed significantly in the MS1 peak table were subjected to MS2 peak annotation. The MetaboAnalystR 4.0 package, including all the curated databases of MetaboAnalyst, was used for MS2 peak annotation.

## RESULTS

### Molecular identification of soybean endophytes based on 16S rRNA gene sequence

**Fig 1:**
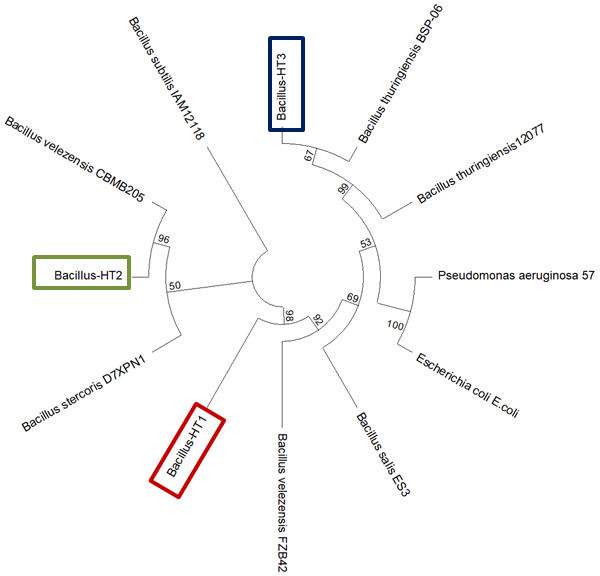
Phylogenetic tree of *Bacillus* based on analysis of 16S rRNA gene sequence of the endophytic strains of soybean.

Based on their differences in colony morphology, three soybean endophytes (HT1, HT2, and HT3) were selected after isolation from soybean roots. Based on 16S rRNA sequencing, we found that these strains belong to the genus *Bacillus* and were thus named *Bacillus*-HT1, *Bacillus*-HT2, and *Bacillus*-HT3 (Fig. 1; Table 1). *Bacillus*-HT1 had 98.94% similarity with *Bacillus velezensis* (NR_075005.2), and *Bacillus-*HT2 had 98.96% similarity with *Bacillus velezensis *(accession no. NR_116240.1), whereas *Bacillus*-HT3 had 99.48% similarity with *Bacillus thuringiensis* (accession no. NR_043403.1). The DNA sequences were aligned, and the phylogenetic tree was constructed by the neighbor-joining method using MEGA5.01 (Fig. 1). The evolutionary history was inferred using the maximum composite likelihood (38). The optimal tree is shown in Fig. 1. The evolutionary distances were computed using the maximum composite likelihood method (39) and are in the units of the number of base substitutions per site. This analysis involved 12 nucleotide sequences. All ambiguous positions were removed for each sequence pair (pairwise deletion option). There was a total of 1,567 positions in the final data set. Evolutionary analyses were conducted in MEGA11 (40). Hence, the 16S rRNA gene sequencing confirmed that the three selected endophytes belong to the genus *Bacillus*.

**TABLE 1:** Closest relative of the soybean endophyte strains as revealed by 16S rRNA gene sequencing

Strain ID	Genus	Length(bp)	Closely related taxa identified by BLAST^*a*^ search		
			Species and strain	Accession number	Highest similarity (%)
HT1	*Bacillus*	567	*Bacillus velezensis* FZB42	NR_075005.2	98.94
HT2	*Bacillus*	759	*Bacillus velezensis* CBMB205	NR_116240.1	98.96
HT3	*Bacillus*	761	*Bacillus thuringiensis* 12077	NR_043403.1	99.48

^*a*^ BLAST, Basic Local Alignment Search Tool.

### Antifungal property

**Fig 2:**
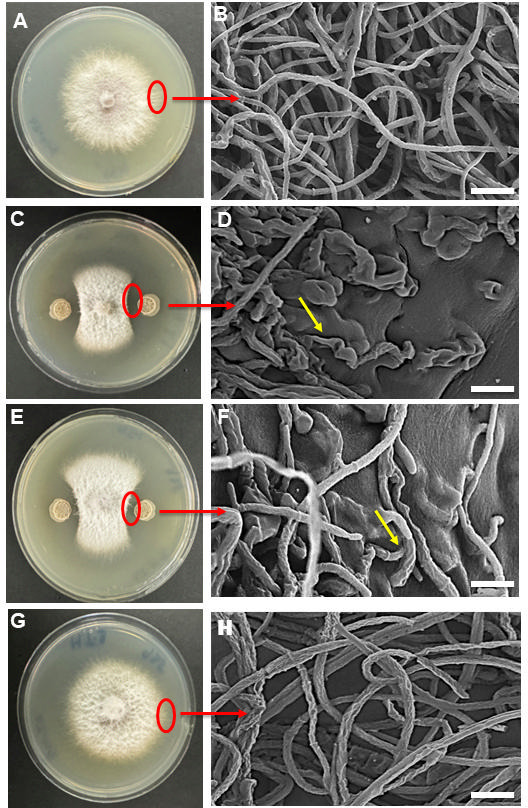
Biocontrol activity of *Bacillus*-HT1, *Bacillus*-HT2, and *Bacillus*-HT3 tested through antagonistic assay. (**A, C, E, and G**) Photographs of petri plates with growing mycelium of *Fusarium oxysporum*. (**B, D, F, and H**) Scanning electron micrographs of fungal hyphae at the edge of the mycelium facing the bacterial culture. (**A and B**) Control without bacteria. (**C and D**) *Bacillus*-HT1, (**E and F**) *Bacillus*-HT2, and (**G and H**) *Bacillus*-HT3. Yellow arrows in panels **D and F** indicate shrinkage of hyphae. Scale bars = 30 µm (**B, D, F, and H**).

Using a dual culture assay, *Bacillus*-HT1, *Bacillus*-HT2, and *Bacillus*-HT3 were tested for *in vitro* biocontrol activity against *F. oxysporum* isolated from soybean. *Bacillus*-HT1 and *Bacillus*-HT2 demonstrated strong antifungal activity against *F. oxysporum in* petri plates. Scanning electron micrographs showed the growth inhibition and shrinkage of fungal hyphae in the presence of *Bacillus*-HT1 or *Bacillus-*HT2 (Fig. 2A through F). *Bacillus*-HT3 did not possess antifungal activity against *F. oxysporum* as it did not inhibit the growth of fungal hyphae (Fig. 2G and H). It was therefore determined that *Bacillus*-HT1 and *Bacillus*-HT2 are both antifungal bacteria, but *Bacillus*-HT3 is not.

### Metabolomics study of secreted bacterial metabolites

Based on their biocontrol activity, we hypothesized that the metabolite profiles of *Bacillus*-HT1 and *Bacillus*-HT2 reflect their antagonistic action against *F. oxysporum*, whereas *Bacillus*-HT3 lacks the metabolites that are involved in this action. The metabolites of bacteria grown in tryptic soy broth were tested in liquid chromatography–tandem mass spectrometry (LC-MS/MS). The spectrally processed data obtained were normalized and analyzed for the following parameters.

#### Multivariate analysis of bacterial exo-metabolomes

**Fig 3:**
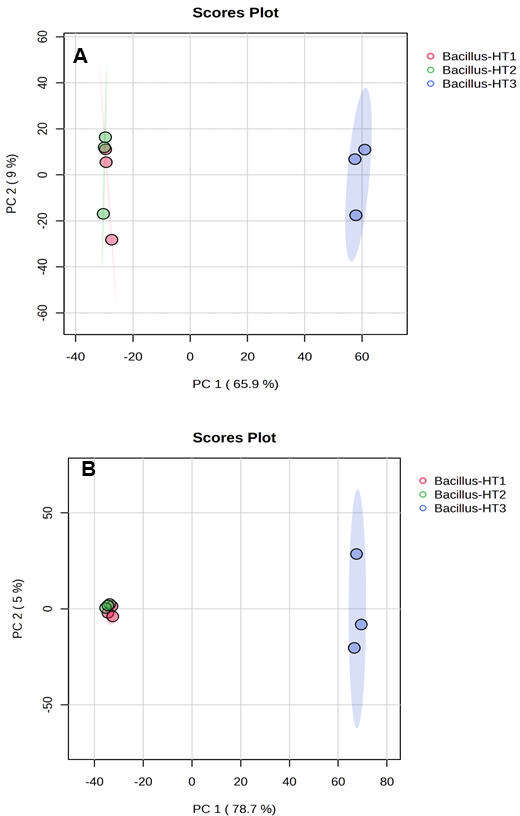
Principal component analysis (PCA) score plot of negative and positive modes of metabolites isolated from *Bacillus*-HT1, *Bacillus*-HT2, and *Bacillus*-HT3. (**A**) PCA features quantified in negative mode having *F* value, 22.962; *R*^2^, 0.88445; *P* value (based on 999 permutations), 0.025. (**B**) PCA features quantified in positive mode. *F* value, 47.059; *R*^2^, 0.94007; *P* value (based on 999 permutations): 0.008.

**Fig 4:**
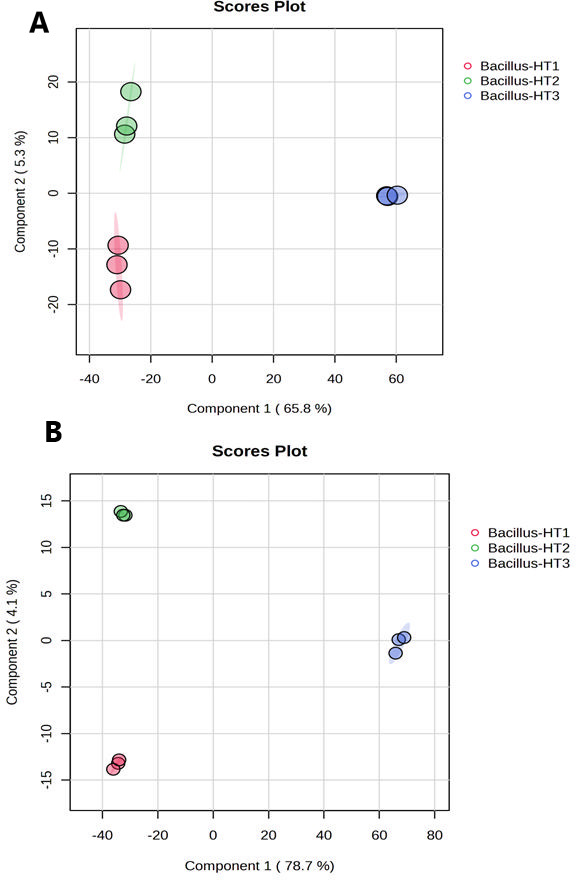
Partial least squares-discriminant analysis (PLS-DA) score plot of negative and positive modes of *Bacillus*-HT1, *Bacillus*-HT2, and *Bacillus*-HT3. (**A**) PLS-DA of features quantified in negative mode. (**B**) PLS-DA of features quantified in positive mode.

Principal component analysis and PLS-DA models were built for the data matrices to observe global trends in the exo-metabolome of different bacteria (Fig. 3 and 4). After raw data processing, a total of 20,583 and 19,495 features were detected and aligned across the whole sample list in negative and positive modes, respectively. To better understand all the information presented in the collected data sets, unsupervised PCA was performed to identify the differences between the sample groups. The PCA score plot showed 65.9% and 9.0% of total variance by PC1 and PC2, respectively, in negative mode (Fig. 3A), and 78.7% and 5.0% by PC1 and PC2, respectively, in positive mode (Fig. 3B). The total variance explained by PCA showed a different distribution of the samples with a high explanation of total variance. Supervised PLS-DA was further performed to identify a small number of linear combinations of the original variables that described most of the variability of the metabolite profile of the three *Bacillus* spp. As presented in Fig. 4, three different clusters corresponding to three different *Bacillus* spp. identified in the PLS-DA score plot for both (+) and (−) ionization modes, in which the first two components cumulatively accounted for 71.1% variation in the negative ionization mode (Fig. 4A) and 82.8% variation (Fig. 4B) in the positive ionization mode, and with the first component explaining (65.8% for negative mode and 78.7% for positive mode) the variation between the *Bacillus* species. PLS-DA clusters the groups more effectively by using a supervised approach, which differentiates between groups, which in the present case are *Bacillus*-HT1, *Bacillus*-HT2, and *Bacillus*-HT3. The PCA and PLS-DA results supply an explanation of the differences in the exo-metabolic profiles of bacteria that correlate with the positive biocontrol activity of *Bacillus*-HT1 and *Bacillus-*HT2 and the absence of biocontrol activity of *Bacillus*-HT3 (Fig. 3 and 4). In addition to this, *Bacillus*-HT1 and *Bacillus-*HT2 showed similarity with *B. velezensis*, which can be attributed to the fact that both can have similar exo-metabolome profile, which is confirmed by PCA groups clustering. The two strains do not have identical exo-metabolome profiles, however, as shown by the clustering of groups in PLS-DA. *Bacillus*-HT3 is different from *Bacillus*-HT1 and *Bacillus*-HT2 in terms of phylogeny and has similarity with *B. thuringiensis*, and it has a different metabolic profile as shown by clustering in PCA and PLS-DA. Therefore, the variable antagonistic activity correlates with differences in the exo-metabolome of three different bacterial strains. The PCA and PLS-DA models provided significant insights into the exo-metabolic profile of bacteria, explaining a high percentage of total variance and highlighting the correlation between the exo-metabolome and the biocontrol activity of *Bacillus* strains.

#### Univariate analysis metabolite

**Fig 5:**
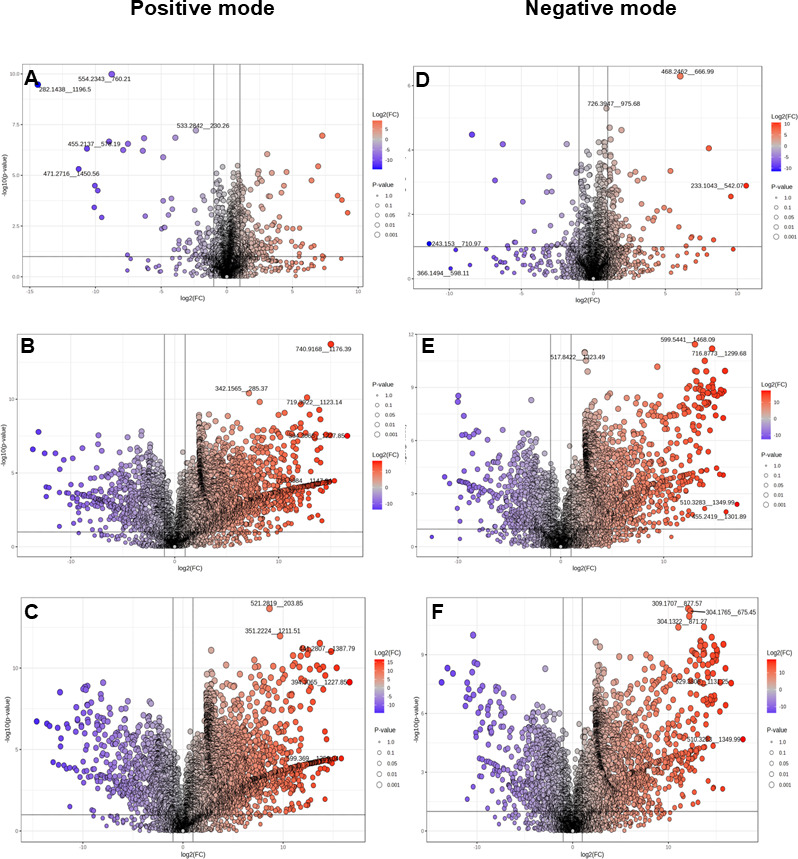
Volcano plots illustrating the distribution of differentially expressed features in the cell-free supernatant of bacteria in positive (**A–C**) and negative (**D–F**) polarities as log10 (*P* values) plotted against log2 (fold change). The black dotted vertical line represents a ±2-fold change. The horizontal line indicates the significance threshold (before logarithmic transformation) at *P* < 0.1. (**A and D**) *Bacillus*-HT1 vs *Bacillus*-HT2, (**B and E**) *Bacillus*-HT1 vs *Bacillus*-HT3, and (**C and F**) *Bacillus*-HT2 vs *Bacillus*-HT3.

Through volcano plots, comparative analysis of variability among the peaks of *Bacillus*-HT1 and *Bacillus*-HT2 (Fig. 5A) revealed 90 significantly upregulated and 217 significantly downregulated peaks in positive mode. However, the case is different when compared with *Bacillus*-HT3. In positive mode, 739 and 2,267 significantly downregulated peaks and significantly upregulated peaks were detected when *Bacillus*-HT1 was compared with *Bacillus-*HT3 (Fig. 5B). Similarly, in positive mode, there were 823 and 2,241 significantly downregulated and upregulated peaks detected in the comparative analysis of *Bacillus*-HT2 with *Bacillus*-HT3 (Fig. 5C). Comparative analysis of *Bacillus*-HT1 and *Bacillus*-HT2 in negative polarity showed 46 downregulated peaks and 149 significantly upregulated peaks (Fig. 5D). In negative mode, 611 and 1,761 significantly downregulated peaks and significantly upregulated peaks, respectively, were detected in the comparative analysis of *Bacillus*-HT1 and *Bacillus-*HT3 (Fig. 5E). Similarly, in negative mode, there were 678 and 1,755 significantly downregulated and upregulated peaks detected in the comparative analysis of *Bacillus*-HT2 and *Bacillus*-HT3 (Fig. 5F). The comparative analysis of *Bacillus*-HT1, *Bacillus*-HT2, and *Bacillus*-HT3 revealed significant features across both positive and negative modes.

#### KEGG pathway analysis of differential metabolites

**Fig 6:**
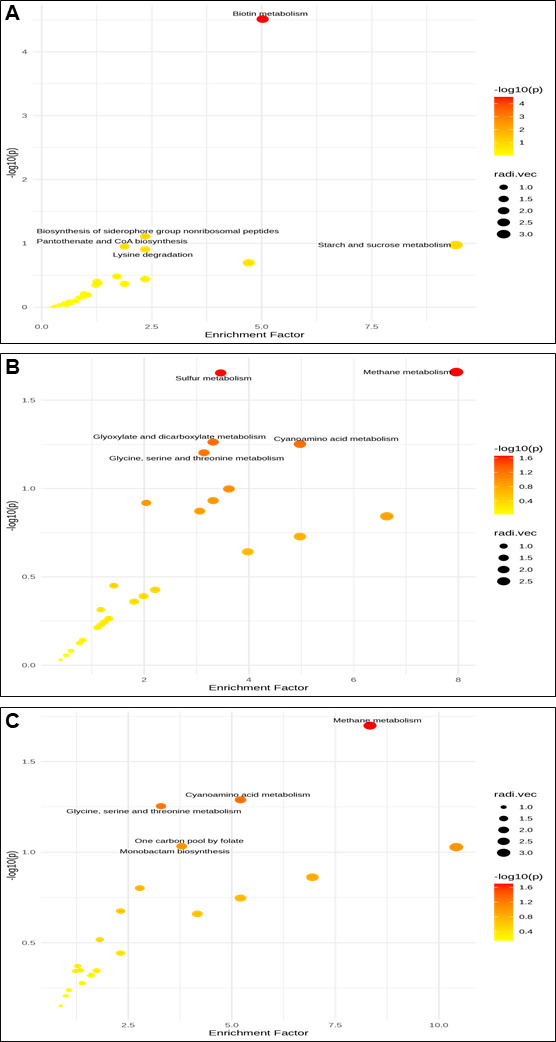
Scatter plot illustrating the enrichment factor of differentially expressed features in positive mode of analysis of cell-free supernatant of bacteria as log10 (*P* values) plotted against enrichment factor (**A**) *Bacillus*-HT1 vs *Bacillus*-HT2, (**B**) *Bacillus*-HT1 vs *Bacillus*-HT3, and (**C**) *Bacillus*-HT2 vs *Bacillus*-HT3.

**Fig 7:**
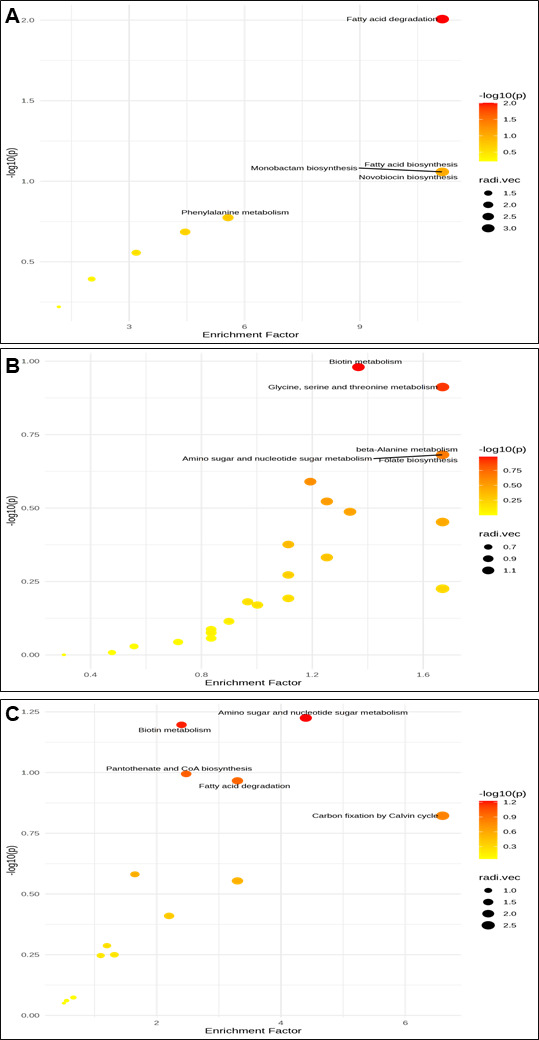
Scatter plot illustrating the enrichment factor of differentially expressed features in negative mode of analysis of cell-free supernatant of bacteria as log10 (*P* values) plotted against enrichment factor. (**A**) *Bacillus*-HT1 vs *Bacillus*-HT2, (**B**) *Bacillus*-HT1 vs *Bacillus*-HT3, and (**C**) *Bacillus*-HT2 vs *Bacillus*-HT3.

The scatter plot (Fig. 6) illustrates the enrichment factor of differentially expressed putative metabolites in positive mode and annotated based on KEGG. KEGG enrichment analysis was performed based on the putative annotation of MS1. The integrative calculation of enrichment factor and −log10 (*P*) of *Bacillus-*HT1 vs *Bacillus*-HT2 indicates the putative metabolites that are involved in different pathways, including biosynthesis of siderophores, biotin metabolism, and pantothenate CoA biosynthesis. Figure 6B shows the *Bacillus*-HT1 vs *Bacillus*-HT3 enrichment factor and indicates the various pathways, including sulfur metabolism and cyanoamino acid metabolism. The differentially expressed metabolites in *Bacillus*-HT2 vs *Bacillus*-HT3 (Fig. 6C) showed peaks involved in various pathways, including monobactam biosynthesis, which confers biocontrol properties to these bacteria. The differentially expressed peaks in *Bacillus*-HT1 vs *Bacillus*-HT2 (Fig. 7A) involved various pathways including novobiocin biosynthesis, monobactam biosynthesis, and fatty acid biosynthesis and degradation. The differentially expressed putative metabolites and their related pathways of *Bacillus*-HT1 vs *Bacillus*-HT3 and *Bacillus*-HT2 vs *Bacillus*-HT3 are shown in Fig. 7B and C. The enrichment factors and −log10 (*P*) values of *Bacillus-*HT1 and *Bacillus*-HT2 indicate their involvement in various metabolic pathways, contributing to their biocontrol properties.

#### Metabolite annotation

The analysis of MS2 spectra and the annotation of metabolites are important aspects of untargeted metabolomics. After the evaluation of the multivariate models, the validated and processed data matrices were used to find biomarkers according to the sample degree of biocontrol activity: *Bacillus-*HT1 = *Bacillus-*HT2 > *Bacillus*-HT3. The peaks that changed significantly in the univariate model were confirmed by MS/MS fragmentation spectra for positive and negative mode (Table S1). The significantly altered antifungal metabolites that are confirmed by MS/MS spectra in positive mode are shown in Table 2. These include important antifungal compounds including bacillibactin, fusidic acid, kynurenine, surfactin C, and geranic acid. The metabolites confirmed by MS/MS spectra in negative mode that have antifungal properties are shown in Table 3. In the present study, MS2 spectral deconvolution was employed using the MetaboanalystR package to obtain high-quality compound identification results for data-dependent acquisition in which contaminated spectra were deconvolved using a database-dependent regression model. The consensus spectra were searched against MetaboAnalyst’s curated MS2 reference databases for compound identification based on dot product or spectral entropy similarity scores as suggested by Pang et al. (41). Searching an unknown spectrum against a database, a similarity score is computed between the query spectrum and database spectra based on the following formula (37, 42):

$\frac{\text{MS2 similarity}\;+\;\text{MS1 similarity}\;+\;\text{retention time similarity}\;+\;0.5\times \text{ isotope similarity}}{3.5}\times 100$

**TABLE 2:** Significantly altered putative antifungal metabolites annotated in the exo-metabolome of the *Bacillus* species (HT1, HT2, and HT3) in positive mode

Groups	Putative compound identification	*m*/*z* value	Fold change	*P* value	Database	Similarity score
*Bacillus*-HT1 vs *Bacillus*-HT2	3,5-Diethyl-2-methylpyrazine	151.1229	0.14	0.02	HMDB predicted	62.02
*Bacillus*-HT1 vs *Bacillus*-HT3	Bacillibactin	883.2625	719.1	0.000236	GNPS	51.03
	Decoquinate	418.2589	5.6	7.68E-06	HMDB_predicted	61.77
	Dehydrocostus lactone	231.1382	6.3	0.02	MoNA	73.66
	Kynurenine	209.0924	0.3	0.000341	MoNA	80.02
	Omega-hydroxydodecanoate	199.1693	6.4	0.0041879	MSDIAL	81.14
	Surfactin C	1,058.6719	11.2	0.0065343	MoNA	72.98
	Telocinobufagin	425.2296	142.78	0.00060681	BMDMS	73.07
*Bacillus*-HT2 vs *Bacillus*-HT3	Bacillibactin	883.2625	756.7	0.00020995	GNPs	51.03
	Beclomethasone 17-monopropionate	465.204	5.1	0.0000000247	HMDB_predicted	57.29
	Fusidic acid	539.3329	6.5	0.0000020634	MoNA	52.5
	Geranic acid	169.1223	0.45	0.052897	MoNA	68.52
	Myriocin	402.2847	15.995	0.000049006	MSDIAL	62.24
	Surfactin C	1,058.6719	6.7	0.013523	MoNA	74.98

**TABLE 3:** Significantly altered putative antifungal metabolites annotated in the exo-metabolome of the *Bacillus* species (HT1, HT2, and HT3) in negative mode

Groups	Putative compound identification	*m*/*z* value	Fold change	*P* value	Database	Similarity score
*Bacillus*-HT1 vs *Bacillus*-HT2	Palmitoleic acid	253.2173	2.3	0.02	MassBank	81.32
	Palmitic acid	255.233	40.8	0.000447	MoNA	86.58
*Bacillus*-HT1 vs *Bacillus*-HT3	Valproic acid	143.1066	5.8	0.000122	HMDB_predicted	67.57
	Linalyl oxide	169.1225	7.9	0.000168	HMDB_predicted	73.97
	Undecanoic acid	185.1537	10.9	0.000021	HMDB_predicted	73.14
	10-Hydroxydecanoate	187.1332	32.9	0.0000012	MoNA	76.63
	12-Hydroxydodecanoic acid	215.1648	29.6	0.000019	MoNA	75.07
	Pentadecanoic acid	241.2171	4.3	0.015959	MassBank	79.26
	3-Hydroxy-tetradecanoic acid	243.1964	17.4	0.000019	HMDB_predicted	67.07
	9-Stearolic acid	279.2331	4.9	0.032455	MoNA	82.87
	7-O-succinyl macrolactin A	501.2497	5.9	0.000031	HMDB_predicted	60.67
*Bacillus*-HT2 vs *Bacillus*-HT3	2-Furoic acid	111.0074	3.4	0.001707	MSDIAL	64.9
	Nonanoate	157.1224	2.1	0.007937	MSDIAL	80.67
	Stearic acid	283.2644	11.7	0.004523	HMDB_experimental	80.82

The similarity score of various putative antifungal metabolites, detected in positive and negative modes, is shown in Tables 2 and 3, respectively. Kynurenine and Surfactin C were produced in significant amounts by *Bacillus*-HT1, having a similarity score of 80.02 and 72.98 with the MoNA database. Table 3 shows the similarity score of various putative metabolites detected in negative mode; e.g., in the exometabolome of *Bacillus*-HT1, palmitic acid was detected and annotated based on the MoNA database, with a similarity score of 86.58. Similarly, a significant amount of stearic acid was present in the exo-metabolome of *Bacillus*-HT2, and it has an 80.8 similarity score compared to the HMDB-experimental database. These findings suggest that *Bacillus*-HT1 and *Bacillus*-HT2 are potent producers of various putative antifungal metabolites, demonstrating high similarity to known compounds in established databases.

## DISCUSSION

Soybean production can be enhanced by controlling its phytopathogens, particularly *Fusarium* spp., including *F. oxysporum*. The pathogen causes soybean root rot, which is a soil-borne disease with a high risk of infection, negatively affecting soybean emergence, seedling growth, plant vigor, and yield losses (43). Some *Fusarium* diseases have been managed using conventional methods, such as seed dressing and leaf spraying with chemical fungicides (44, 45). Considering the adverse effects of chemical fungicides on the environment, human health, and pathogen resistance, increased attention has been focused on the limitations of control by way of synthetic chemicals (46, 47). Biological control of plant diseases using plant-associated microbiota and natural compounds of biological origin is becoming increasingly accepted as an alternative to the use of chemical fungicides (48). This study focuses on the use of soybean endophytes against *F. oxysporum* and explores the mechanism behind this biocontrol activity by analyzing the metabolites secreted by these endophytes. Endophytic bacteria receive considerable attention and may prove to be more important than rhizobacteria in supporting plant growth and health because they are in closer contact with their host plants and can, in addition to having antifungal activities, directly increase plant tolerance to biotic and abiotic stress factors (49). This study describes the isolation of three *Bacillus* spp. from soybean plant roots and screens against *F. oxysporum* in *in vitro* confrontation assays, which revealed antagonistic activity for *Bacillus-*HT1 and HT2 against *F. oxysporum*, whereas *Bacillus*-HT3 did not display any growth-inhibiting activity against mycelium growth. The inhibitory action of *Bacillus*-HT1 and *Bacillus*-HT2 was visible both at macroscopic and microscopic levels, with fungal hyphae displaying shrinkage when confronted with the bacteria. This is consistent with fungal responses to other endophytes like *Pseudomonas aeruginosa* OS_12 and *Aneurinibacillus aneurinilyticus* OS_25, *Paenibacillus polymyxa*, *B. aminoliquefaciens* HT, and *B. velezensis* BQ, which were shown to significantly inhibit the growth of *F. oxysporum*. When examined by SEM, the hyphae in the confrontation zones were characterized as heavily swollen, wrinkled, collapsed, and with cracks (50, 51).

LC-MS techniques provide an effective platform for chemical screening, comparison, and validation of metabolites produced by bacterial fermentation, which has promising applications in sustainable agriculture (52, 53). The untargeted metabolomics data of the present study, after univariate and multivariate analyses, confirmed that *Bacillus*-HT1 and *Bacillus*-HT2 have similar metabolomic profiles that we attribute to their antagonistic activity against *F. oxysporum* and may also reflect their phylogenetic similarity. The metabolic profile of *Bacillus*-HT3 differs from the other two, consistent with the lack of inhibitory action on the growth of *F. oxysporum*. The enrichment factor based on KEGG annotation confirmed that the putative metabolites produced by these bacteria are involved in antibiotic pathways (novobiocin synthesis and monobactam biosynthesis) and siderophore biosynthesis. Cao et al. (54) reported that the microbial inoculant *Bacillus atrophaeus* DX-9 is antagonistic to potato common scab, and a metabolomics study demonstrated that it can significantly reduce potato scab disease and increase soil levels of phytolaccoside A, 7,8-dihydropteroic acid, novobiocin, and azafrin. DNA gyrase and topoisomerase IV are validated targets for the development of dual-targeting antibacterial agents. Aminocoumarins (such as novobiocin and clorobiocin) are therapeutically relevant antibacterial drugs that target both enzymes (55). Other researchers similarly reported that the suppression of plant-pathogenic fungi by *Bacillus amyloliquefaciens SB-1*, *Bacillus subtilis* A-2, and *Bacillus tequilensis* A-3 is mediated by their secondary metabolites (56). Our findings are consistent with these studies on various bacterial strains and corroborate the potential of antifungal compound-producing bacteria as promising biocontrol agents.

The number of peaks that were significantly altered in univariate analysis in the present study was subjected to MS2 annotation. An analysis of the gene clusters of *Bacillus* sp. revealed that its genes are involved in the non-ribosomal synthesis of cyclic lipopeptides such as surfactin, bacillomycin, fengycin, and bacillibactin (57, 58). Surfactants are antibiotic compounds that alter the integrity of membranes (59). According to Liu et al. (60), surfactin-producing *B. subtilis* inhibits the growth of *Fusarium foetens* (closely resembling *F. oxysporum*) using various mechanisms, resulting in malformed mycelial morphology, including alterations to cell membrane permeability, lysis of cell contents, induction of differential protein expression, changes in cell metabolic pathways, and binding to DNA in groove-binding mode, leading to inhibition of replication or transcription. This is consistent with our findings that surfactin-producing *Bacillus* spp. (HT1 and HT2) inhibit the growth of *F. oxysporum* and cause mycelial malformation. Bacillibactin D has an array of antibacterial and antifungal properties, as well as hemolytic properties (61). Additionally, bacillibactin is thought to possess antimycobacterial activity against plant pathogens (62). In the present study, based on the MS2 fragmentation similarity score with the database, we found that *Bacillus*-HT1 and *Bacillus*-HT2 produce pronounced levels of putative metabolites, including bacillibactin and surfactin C, which are likely to contribute to their biocontrol activity against *F. oxysporum*. Kumar et al. (63) confirmed the potent antagonistic activity of bacillibactin against phytopathogens *Alternaria porri* and *Fusarium equisetiporri*, supporting its potential as a promising biological control agent for fungal plant diseases as it causes fungal biomass reduction, elevated antioxidative enzyme levels, and membrane damage to fungal structure. Bacillibactin is a siderophore and non-ribosomal peptide that acts as an antimycobacterial agent and has been reported as antibacterial against the plant pathogen *Pseudomonas syringae* and antifungal against *Verticillium dahliae*, *Fusarium oxysporum*, *Aspergillus flavus*, and *Rhizoctonia solani* (64). Microbiome–host cell interactions result in the production of various bioactive compounds, including kynurenine derivatives, that have profound effects on both local and systemic processes. *Lactobacillus reuteri*, for instance, preferentially synthesizes kynurenic acid from kynurenine to alleviate gastrointestinal pathogens (65, 66). Here, kynurenine was detected as a putative metabolite produced by *Bacillus*-HT1 having a similarity score of 80.02 with the MoNA database, which can be attributed to the fact that *Bacillus*-HT1 is an antifungal bacterium that can potentially inhibit fungal growth.

Saturated fatty acids have been reported to exert inhibitory activity toward some fungal species (67, 68). Other researchers reported that palmitoleic acids can inhibit some fungal growth, particularly *Candida albicans* (69). In the present study, we found a significant amount of palmitoleic acid and palmitic acid in the exo-metabolome of *Bacillus*-HT1 with similarity scores of 81.32 and 86.58 with the MassBank and MoNA databases, respectively, which explains why this bacterium can be used for broad-spectrum purposes to control fungal diseases of plants. Similarly, *Bacillus*-HT1 produced significant putative antifungal fatty acids (valproic acid, undecanoic acid, 10-hydroxydecanoate, hydroxydodecanoic acid, pentadecanoic acid, 3-hydroxy-tetradecanoic acid, and 9-stearolic acid), and *Bacillus-*HT2 produced stearic acid, while *Bacillus-*HT3 did not produce any of these fatty acids. This is consistent with the strong antifungal potential of *Bacillus*-HT1 and HT2 against *F. oxysporum*. Similarly, Korany et al. (70) reported that tridecanoic acid, pyrrole, and pentadecanoic acid are different types of fatty acids producing *B. atrophaeus* SM3 that are antagonistic against fungal pathogens, including *F. oxysporum*, and inhibit mycelium growth and cause cell shrinking and alterations in the cell wall thickness when visualized by transmission electron microscopy. Macrolactins have been described as an important group of 24-membered macrolides mainly produced by *B. subtilis*, with most of them principally having antibacterial and antifungal activities (71, 72). Here, we found that *Bacillus-*HT1 produces a significant amount of putative 7-O-succinyl macrolactin and similarity score of 60.67 compared to the HMDB database, which predicted its putative identity compared to *Bacillus-*HT3. Salazar et al. (73) found that 7-O-succinyl macrolactin inhibits the growth of *F. oxysporum* and *Moniliophthora roreri* and causes swelling and damage to fungal hyphae and reduced formation of reproductive structures. Based on LC-MS/MS and the results of MS2 annotation, we found that *Bacillus*-HT1 and *Bacillus*-HT2 secrete putative antifungal metabolites, supporting the notion of their antifungal potential. As endophytes isolated from soybean, both strains are likely to be plant-beneficial bacteria that can control phytopathogens. *Bacillus*-HT3 lacks this antifungal activity. However, as a member of the endophyte community of soybean, this bacterium might be able to stimulate plant growth directly or inhibit other pathogenic bacteria or fungi, but further research is needed to explore this potential.

### Conclusion

Plant endophytes are beneficial communities of bacteria that produce phytohormones, antifungal metabolites, and siderophores, induce systemic resistance against phytopathogens, and play a crucial role in sustainable crop production. The present study explored the biocontrol potential of isolated endophytes using metabolomics to uncover the metabolites that might mediate the beneficial effects. We showed that these bacteria produce antifungal metabolites such as surfactin, bacillibactin, fusidic acid, and certain fatty acids that probably mediate inhibitory effects on the fungal growth of *F. oxysporum*. To explore the other potentially beneficial effects of these bacteria, further omics studies are required by testing the role of these bacteria in eliciting increased resistance in soybean and other plants under stress conditions. Future research should integrate transcriptomics and genomics to gain insights into the gene expression profiles and genetic makeup of soybean endophytes. Additionally, proteomics could be employed to study the protein interactions and pathways involved in the biocontrol mechanisms. Combining these omics approaches will provide a comprehensive understanding of the symbiotic relationship and enhance the development of sustainable agricultural practices.

## Data Availability

The 16S rRNA nucleotide sequences of *Bacillus*-HT1, *Bacillus*-HT2, and *Bacillus*-HT3 were submitted to GenBank under accession numbers PV534845, PV534846, and PV534847. The data sets presented in this study can be found in online repositories. The names of the repository/repositories and accession numbers can be found at CCMS MassIVE (accession no. MSV000097347).

## SUPPLEMENTAL MATERIAL

Abstract msystems.00927-25-s0001.docx Graphical abstract.

Table S1 msystems.00927-25-s0002.xlsx MS2 annotation of significantly upregulated metabolites detected in positive mode and negative mode.

ASM does not own the copyrights to Supplemental Material that may be linked to, or accessed through, an article. The authors have granted ASM a non-exclusive, world-wide license to publish the Supplemental Material files. Please contact the corresponding author directly for reuse.
