# Supplementary figures and images for: Exo-metabolome profiling of soybean endophytes: a road map of antagonism against Fusarium oxysporum
Source: mSystems. 2025 Oct 1;10(10):e00927-25. doi: 10.1128/msystems.00927-25 (PMC12542665; doi:10.1128/msystems.00927-25)

**Graphical Abstract**

**
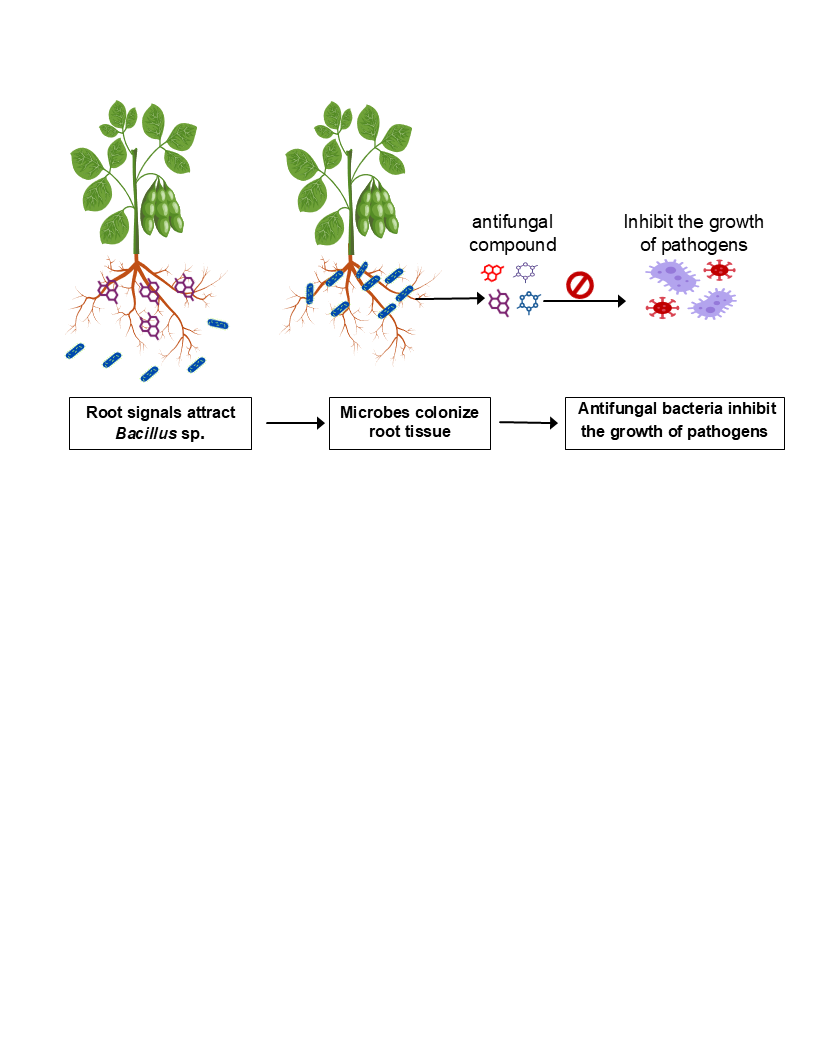
**

Supplement: Abstract — Graphical abstract. [file msystems.00927-25-s0001.docx]
